# Functionalization of graphene at the organic/water interface†
†Electronic supplementary information (ESI) available. See DOI: 10.1039/c4sc03504f



**DOI:** 10.1039/c4sc03504f

**Published:** 2014-11-25

**Authors:** Peter S. Toth, Quentin M. Ramasse, Matěj Velický, Robert A. W. Dryfe

**Affiliations:** a School of Chemistry , University of Manchester , Oxford Road , Manchester M13 9PL , UK . Email: robert.dryfe@manchester.ac.uk ; Tel: +44 (0)161-306-4522; b SuperSTEM Laboratory , STFC Daresbury Campus , Daresbury WA4 4AD , UK

## Abstract

A simple method for the deposition of noble metal (Pd, Au) nanoparticles on a free-standing chemical vapour deposited graphene (CVD GR) monolayer is reported. The method consists of assembling the high purity CVD GR, by transfer from poly (methyl methacrylate) (PMMA), at the organic/water interface. Metal deposition can then proceed using either spontaneous or electrochemically-controlled processes. The resultant graphene-based metal nanoclusters are characterized using atomic force and electron microscopy techniques, and the location of the nanostructures underneath the graphene layer is determined from the position and the intensity changes of the Raman bands (D, G, 2D). This novel process for decoration of a single-layer graphene sheet with metal nanoparticles using liquid/liquid interfaces opens an alternative and useful way to prepare low dimensional carbon-based nanocomposites and electrode materials.

## Abstract 2

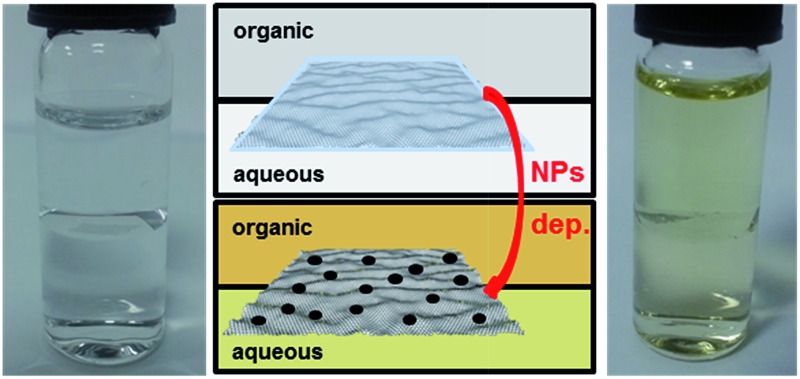
A simple method for the deposition of noble metal (Pd, Au) nanoparticles on a free-standing chemical vapour deposited graphene monolayer is reported. Metal deposition can proceed using either spontaneous or electrochemically-controlled processes. The resultant nanoclusters are characterized using atomic force and electron microscopy techniques, and mapping mode Raman spectroscopy.

## Introduction

The combination of carbon, as a conducting support material, with metal nanoclusters is of primary importance for developments in the field of catalysis, and more specifically, in electrocatalytic processes such as CO_2_ reduction, H_2_ evolution and water oxidation.^1–3^ Traditional porous carbon materials are being replaced with low-dimensional forms of carbon and graphitic nanostructures, *e.g.* carbon nanotubes,^3^ fullerenes^2^ and exfoliated graphite,^3^ to develop novel electrode materials for improved energy conversation. In particular, the two dimensional allotrope of carbon, graphene, and its single atomic thick structure, has attracted great attention due to its unique properties (electronic structure, inherent strength, transparency) since its first isolation.^4^ Several preparation methods, producing different size flakes and grades of graphene (exfoliation,^4^ chemical vapour deposition, CVD,^5,6^ reduced graphene-oxide (rGO) prepared in solution^7^) have been described recently. Subsequently, there has been great interest in graphene functionalization,^8–10^ or doping/charging with metal nanoparticles (NPs) and the formation of larger structures.^11–16^ The functionalization or doping of carbon nanomaterials can be characterised by Raman spectroscopy.^8–10,13,15,17^ The Raman spectrum of single-layer graphene sheets has two characteristic peaks (G and 2D bands) due to the carbon lattice; the G peak is associated with the E_2g_ vibration mode of the sp^2^ framework and the 2D band is caused by the scattering of two phonons and although independent of defects, it is instead affected by strain.^17–19^ Graphene is conventionally supported on solid surfaces, such as Cu films used as growth substrates during the CVD process, or transferred to other solids such as polymers or oxide-covered silicon wafers, to enable lithographic processing or optical characterization, respectively. One issue that has been widely recognised is the extent to which solid substrates dope the graphene layer.^6,12,13,20^ There is on the other hand a far smaller body of work on the localization of graphene at the liquid/liquid interface. One advantage of interfacial assembly of graphene is the ability to spontaneously form macroscopic films from self-assembly of individual flakes.^21–24^ This approach has been exploited for flakes (3–4 nm thick) of multilayer graphene,^23^ for rGO materials,^22^ and for a mixture of monolayer and few-layer graphene^24^ derived from solution phase exfoliation. However, we are unaware of any prior reports of assembly of individual, macroscopic (cm^2^ scale) graphene flakes at the liquid/liquid interface. Furthermore, when both liquid phases contain an electrolyte, the interface between two immiscible electrolyte solutions (ITIES) can be electrically polarized to generate electrochemical potential gradients that are capable of promoting ion and electron transfer across the molecular boundary, the potential drop across the liquid/liquid boundary being developed over a region of sub-micron thickness.^25–27^ The structure of this interface has been studied: molecular dynamics simulation predicted locally sharp points at the interface roughened by capillary waves,^28,29^ consistent with the experimentally observed electrical capacitance, which exceeded the value predicted by the Gouy–Chapman theory.^30^ For instance, the measured interfacial width in the case of the water/2-heptanone interface was found to be 7.1 Å by X-ray reflectivity, which compared favourably with the calculated value from capillary wave theory of 7.3 Å.^31^ Conventionally, the ITIES is formed from a denser organic solution (usually halogenated or aromatic, and often toxic) and an upper aqueous solution. The formation and assembly of metallic structures, and associated catalytic activity, *i.e.* hydrogen and oxygen evolution, at the organic/water interface has attracted considerable interest in the last decade.^32–34^ The liquid/liquid interface offers the possibility of incorporating, as well as constructing *in situ* at the interface, highly complex catalytic centres and supports, including photoactive oxides^35^ and metallic nanostructures.^36,37^ Low dimensional carbon species, such as carbon nanotubes and multilayer graphene, and electrostatically stabilised nanostructures can be reversibly assembled at the polarisable liquid/liquid interfaces.^35^ Polymer–graphene composites have been formed from assembly of rGO films by polymerization of aniline at the water/toluene interface.^38^ Using easily treatable rGO flakes has led to elegant work on the deposition of noble metals at oil/water-interface-assembled-rGO, mainly to prepare graphene-based catalysts or electrode materials.^15,39–41^ Specifically, the Suzuki–Miyaura cross-coupling reaction was catalysed by Pd NP thin film coated rGO flakes,^40^ while using the same approach Pd, Ag and Au NPs containing films were deposited at the interface from the organic phase to prepare catalyst materials for reduction of nitroaromatic compounds.^15^ The electrocatalytic activity, with respect to methanol oxidation, of Pt/Pd bimetallic thin film covered rGO was reported.^39^ The development of a new type of flexible electrochemical biosensor based on graphene “paper” loaded with Au@Pt core shell nanoparticles to sense nitric oxide has also been reported.^41^

Herein a reverse ITIES configuration is employed, consisting of a lower toxicity, non-halogenated, non-aromatic lower density solvent, which of course must retain its low mutual solubility with water.^42–44^ This is used to illustrate a very simple strategy to prepare new generation catalyst materials, *i.e.* GR monolayers decorated with noble metal nanostructures, Pd and Au NPs, using macroscopic, monolayer CVD graphene (as opposed to rGO) as the substrate. A number of microscopic and nano-scale characterization techniques are applied to graphene-based metal nanoclusters to identify their structure and morphology. In particular, mapping mode Raman spectroscopy is the appropriate technique to confirm the deposited NPs and nanostructures' location, which is shown to be underneath the single-layer graphene.

## Experimental

### Materials

Lithium chloride (LiCl, 99.99%); lithium perchlorate (LiClO_4_, ≥99.5%); tetrabutyl ammonium perchlorate (TBAClO_4_, ≥99.0%); 1,1′-dimethylferrocene (DMFc, 97%); ammonium tetrachloropalladate ((NH_4_)_2_PdCl_4_, 99.995%); potassium tetrachloroaurate (KAuCl_4_, 98%); bis(pentamethylcyclopentadienyl)iron(ii) (Me_10_FeCp_2_, DecMFc, 97%) were purchased from Sigma-Aldrich. The copper foil was removed by etching with ammonium persulfate (98%, Lancaster Synthesis Ltd); 3% 950 K PMMA in anisole (MicroChem Corp.) was used for graphene transfer. The organic phase electrolyte, bis(triphenylphosphoranylidene)ammonium tetrakis(4-chlorophenyl)borate (BTPPATPBCl), was prepared as described elsewhere^35^ from potassium tetrakis(4-chlorophenyl)borate (KTPBCl, ≥98.0%, Sigma-Aldrich) and bis(triphenylphosphoranylidene)ammonium chloride (BTPPACl, 97%, Sigma-Aldrich). The organic phase was formed from mixtures of 1,2-dichloroethane (DCE, ≥99.8%) and di-*n*-butyl ketone (5-nonanone, 98%) purchased from Sigma-Aldrich and used as received. Deionized water (18.2 MΩ cm resistivity), purified by a “PURELAB” Ultrafiltration unit (Elga Process Water, Marlow, UK), was used for aqueous solution preparation. Glassware was cleaned in Piranha solution, a 1 : 4 mixture (by volume) of 30% hydrogen peroxide (H_2_O_2_, Fisher Scientific) and concentrated sulphuric acid (H_2_SO_4_, Fisher Scientific) – CAUTION required when handling – boiled in ultra-pure water and dried.

### CVD graphene growth and transfer

The CVD graphene was a gift from the Bluestone Global Technology (Manchester, UK) and was prepared using methods previously reported.^45^ The monolayer graphene films were transferred from the Cu foil onto Si/SiO_2_ substrates. The transfer was carried out by spin-coating the CVD graphene with 3% 950 K PMMA solution (in anisole) on the Cu foil at 3000 rpm for 60 s using a SPIN200i-NPP spin processor (SPS-Europe B.V., The Netherlands). This resulted in the formation of a PMMA/CVD GR/Cu foil sandwich structure. The Cu foil was etched from underneath the PMMA/CVD GR multilayer by 0.5 M persulphate solution and the remaining flake was “fished out” with a Si/SiO_2_ wafer, and then cleaned three times by transfer to pure water, resulting in a pure PMMA/CVD GR multilayer (“pure”, indicates without residual copper).

### Pristine and decorated CVD GR characterisation

Raman spectroscopy was carried out using a Renishaw RM MkI1000 (633 nm) and a Renishaw RM 264N94 (532 nm) spectrometer operating at power ≤1 mW. The SEM (Philips XL30 ESEM-FEG, operated at 15 kV), the AFM (Bruker MultiMode 8, operated in “Peak Force” tapping mode with a silicon tip on a silicon nitride lever) characterization was performed on the samples once transferred to a Si/SiO_2_ wafer. Further characterization was carried out by Scanning Transmission Electron Microscopy (STEM, Nion Ultrastem™ 100, SuperSTEM, Daresbury, UK), using specifically prepared free-standing samples supported on lacey carbon-coated copper grids (TAAB Laboratories Equipment Ltd). The microscope instrument is equipped with a cold-field emission electron gun with a native energy spread of 0.35 eV and was operated with a beam energy of 60 kV (below the carbon knock-on threshold) and in near-ultra-high vacuum (below 2 × 10^–9^ Torr near the sample) to prevent any damage to the sample (and the graphene in particular) through electron-beam interactions or chemical etching. The probe-forming optics were configured to provide a beam convergence semi-angle of 30 mrad, corresponding to a probe size of approximately 0.11 nm. An estimated electron beam current of about 20 pA was impinging on the sample. STEM images were acquired in high-angle annular dark field (HAADF) mode with the detector inner and outer radii being calibrated at 85 mrad and 190 mrad, respectively. In these conditions, the image intensity is, to a good approximation, proportional to ∼*Z* ^2^ (where *Z* is the atomic number of the object being imaged) and the HAADF mode is thus often referred to as “*Z*-contrast” imaging. EEL spectra were recorded using a Gatan Enfina spectrometer, with a collection semi-angle of 37 mrad.

### Electrochemical techniques

Cyclic voltammetry for the electrochemical deposition of Pd NPs and electrochemical impedance spectroscopy (EIS) to investigate the graphene monolayer stabilisation effect were carried out using a four-electrode configuration (see ESI-1†) with an Autolab PGSTAT302N potentiostat (Metrohm-Autolab, Utrecht, The Netherlands). The cells used for the liquid/liquid electrochemical measurements had an interfacial working area of 0.74 cm^2^ and a total solution volume of 2.5 mL.

## Results and discussion

### CVD GR assembly at the organic/water interface

**Fig. 1:**
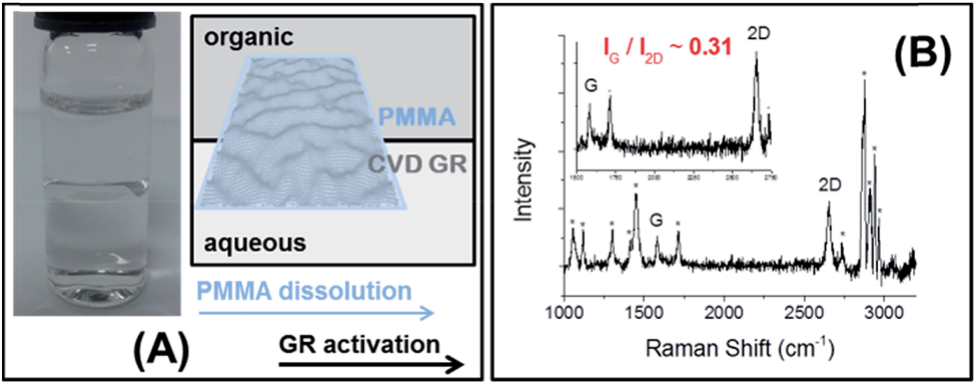
Photograph, schematic (A) and Raman spectrum (B, 633 nm) of assembled PMMA/CVD GR multilayer at the liquid/liquid interface, the inset of the Raman spectrum shows the magnification of the G and 2D bands. The organic phase is pure 5-nonanone.

As described above, the graphene monolayer was grown on copper foil by CVD,^5,6^ spin-coated with *ca.* 100 nm thick PMMA, then the copper was etched from the graphene to yield a PMMA/CVD GR multilayer floating on the aqueous etching solution surface, as the hydrophobic graphene material can float on any water surface.^23,46^ The pristine single-layer graphene was characterised *via* microscopy (see ESI-2†) and the thickness before and after the PMMA spin coating was determined by atomic force microscopy (AFM) (see ESI-3†). The PMMA/CVD multilayer was easily transferred, using thermally oxidized silicon wafers (290 nm thick layer of silicon dioxide, SiO_2_), Si/SiO_2_, to the surface of an aqueous electrolyte solution (0.1 M LiClO_4_). A lower density organic electrolyte solution (0.1 M TBAClO_4_) was then placed on top of the aqueous phase, forming an ITIES modified with the assembled PMMA/CVD GR (Fig. 1A photograph and schematic). This floating square can be seen at the interface in the “inverse” ITIES configuration, where the less dense (upper) organic solvent was 5-nonanone. To enable the dissolution of the PMMA overlayer, parallel experiments were performed where the organic phase, placed in contact with the PMMA/CVD GR, was a DCE/5-nonanone mixture, which enables a much more rapid dissolution of the PMMA than the pure 5-nonanone phase.

This assembly of CVD GR at the organic/water interface has not been reported previously, to the best of our knowledge, and the high quality of the monolayer sample was proven with *in situ* Raman spectroscopy at the liquid/liquid interface (Fig. 1B, *I*_D_/*I*_G_ = 0.31 at 633 nm excitation wavelength). The spectrum (633 nm) depicts the G and the 2D bands of the graphene: the peaks with an asterisk denote the 5-nonanone Raman signals,^47^ the reasonable intensity of the G and 2D bands are shown in the inset, which could open the possibility for *in situ* Raman spectroelectrochemistry of interface-assembled graphene. The intensity ratio of the G and 2D bands (*I*_G_/*I*_2D_) was found to be 0.31, which is close to the ratio of 0.24 from 488 nm excitation^48^ and the full width at half maximum (FWHM) of the 2D band is 30.4 cm^–1^, which is consistent with undoped single-layer graphene (when the Fermi energy is exactly at the Dirac point).^17,18^

**Fig. 2:**
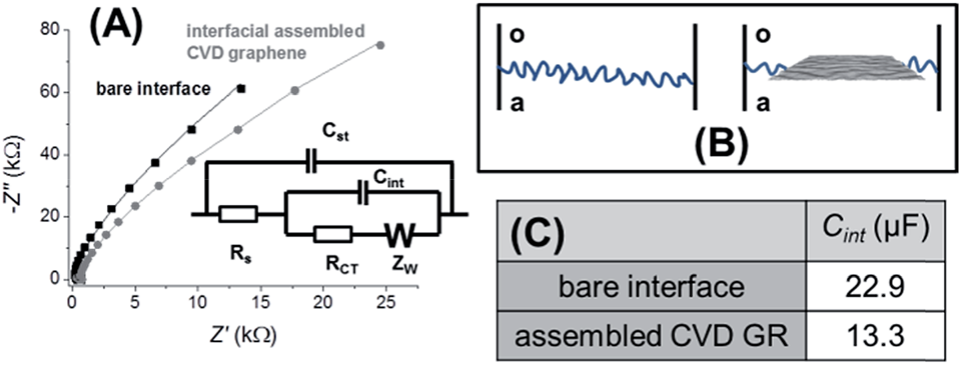
The impedance spectra at –0.1 V (with the equivalent circuit) show the difference between the two interfaces (A), schemes of the bare interface (B, left) and the CVD GR layer stabilized interface (B, right), the measured capacitance values are displayed in the table (C). The DCE/5-nonanone ratio was 1 : 1.

Another feature of the location of CVD GR at the ITIES is its likely suppression of capillary waves which locally roughen the interface.^28,30^ Electrochemical impedance spectroscopy was carried out with only the supporting electrolytes in both phases at different potential differences (–0.3 V, –0.1 V and +0.1 V). The impedance data were analysed and fitted using the equivalent electrical circuit^49,50^ which is depicted by Fig. 2A, where *C*_int_ is the capacitance of the interface, *R*_CT_ is the charge transfer resistance, *Z*_W_ is the Warburg impedance, *C*_st_ is the stray capacitance and *R*_s_ is the solution resistance. The complex impedance Nyquist plots at –0.1 V from the bare (Fig. 2B, left-hand schematic) and the assembled CVD GR (Fig. 2B, right-hand schematic) interfaces are shown in Fig. 2A. The graphene layer coverage at the interface was estimated to be 50%, *i.e.* 0.35 cm^2^ compared to a total contact area of 0.74 cm^2^ (the dimensions of the CVD GR single-layer flake were 5 mm by 7 mm). The interfacial capacitance was decreased with the single-layer graphene at the interface (see table in Fig. 2C), which is interpreted as the capillary wave roughness of the bare interface (Fig. 2B left) having a higher capacitance (analogous to a porous material), than the case of the CVD GR monolayer stabilised interface (Fig. 2B right).

### Spontaneous decoration of the CVD GR single-layer

**Fig. 3:**
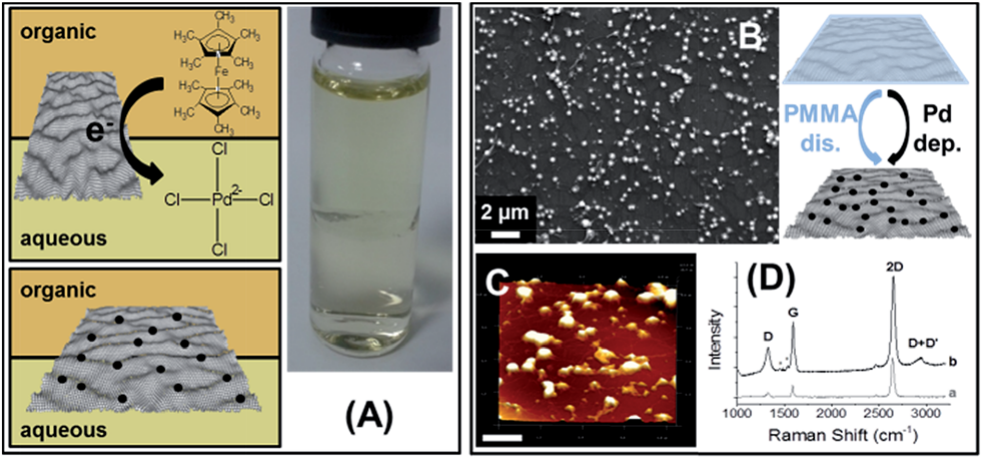
(A) Schemes of the PdCl_4_^2–^ reduction by DecMFc *via* CVD GR at the interface (above) by forming the black dots marked Pd NPs (below), shown on the photograph, and the Raman spectra (D, 633 nm) of the pristine and Pd-deposited CVD GR. SEM (B) and AFM (C, scale bar: 1 μm) images and schematics of the Pd NPs decorated CVD GR layer, when the metal deposition and the dissolution of the PMMA takes place simultaneously. The interfacial contact time of deposition was 1 min in the all cases. The DCE/5-nonanone ratio was 1 : 4.

Functionalization of the interface-assembled high purity CVD GR was achieved by a simple spontaneous redox reaction between decamethylferrocene (DecMFc), which acts as an organic phase electron donor, and tetrachloropalladate (PdCl_4_^2–^) in the aqueous phase^32^ (reaction 1). This metal deposition reaction can proceed spontaneously at the liquid/liquid interface, but in the presence of the graphene monolayer, it is found to occur preferentially on the graphene. A schematic of the redox process and a photograph of the Pd decorated CVD GR layer (hereafter, Pd-CVD GR) at the interface are shown in Fig. 3A.

1 PdCl_4_^2–^ (aq) + 2DecMFc (o) → Pd (s) + 4Cl^–^ (aq) + 2DecMFc^+^ (o)

The Raman spectra (633 nm excitation) of pristine and Pd-deposited CVD GR transferred to the Si/SiO_2_ wafer, after 1 min of deposition at the interface, are also shown in Fig. 3D. Shifts of the G, D and 2D bands are observed, which indicates either strain or a functionalization/doping effect on the graphene.^8,9,17^ The width of the 2D band increases to 42.1 cm^–1^ verifying that doping of the CVD GR, due to the presence of Pd, occurs.^9,51^

In the above case, the organic solvent ratio was 1 : 4 (by volume DCE/5-nonanone) to allow rapid dissolution of the PMMA from the top of the CVD GR. The question is, what is happening at the interface? Assuming that the PMMA dissolution and the Pd NPs deposition are taking place simultaneously, as in this system the best conductor is the graphene monolayer,^4,52^ so the deposition starts due to the activation of the graphene, as the PMMA dissolves. The Pd-CVD GR samples were therefore transferred to Si/SiO_2_ for further characterization. Schematics of the dissolution and deposition processes, scanning electron microscopy (SEM) and three dimensional AFM images of the samples after 1 min of deposition are presented in Fig. 3B–C. The deposits are globular, with size distributions mainly lying in the range of 40–300 nm, and these are deposited preferentially at the grain boundaries of the CVD GR: PMMA has been dissolved first from the topographically higher grain boundaries (2–10 nm, see ESI-2†), however there may also be a preference for metal deposition on these boundaries. An approximate kinetic comparison of spontaneous Pd NP deposition at the bare and CVD GR modified interface demonstrates that the metal deposition occurs preferentially on the graphene: as a function of the deposition contact time, the graphene layer becomes darker after 1 min, while in the case of the bare interface the dark Pd layer can be seen only after 15 min, which suggests that spontaneous Pd deposition is faster on the CVD GR than on the bare interface (see also ESI-4†).

**Fig. 4:**
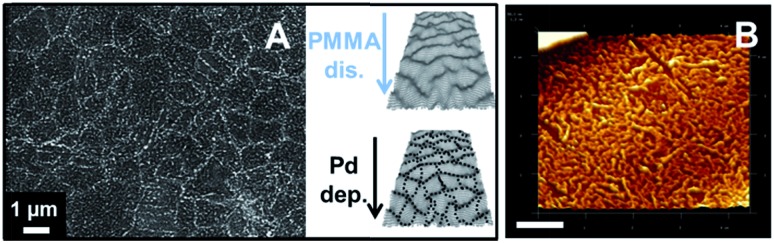
SEM (A) and AFM (B, scale bar: 1 μm) images of the Pd NPs decorated CVD GR layer when the PMMA dissolution is carried out before the NP deposition. The interfacial contact time of deposition was 1 min. The DCE/5-nonanone ratio was 2 : 3 in the organic phase.

In order to separate the PMMA dissolution process from the metal deposition process, the PMMA was dissolved first from the top of the assembled CVD GR single-layer (DCE/5-nonanone volume ratio of 2 : 3), and when the graphene became invisible at the ITIES, the organic phase of the cell was replaced with the solution containing the DecMFc reducing agent: a dark layer was immediately observed to form at the interface, corresponding to the Pd NPs decorating the CVD GR. The schemes of the prior PMMA dissolution and subsequent deposition, the SEM and three dimensional AFM images are shown in Fig. 4. The difference is clearly seen on the SEM image (Fig. 4A), the NPs can be seen everywhere on the sample, both on the grain boundaries (brighter lines) and on the planes, with around 0.24 relative Pd metal content (Pd/C). The AFM image (Fig. 4B) shows line-like structures, containing the Pd NPs in the 20–40 nm size range. The formation mechanism of the two different kinds of deposited metal nanostructures can be understood as a function of the timing of the PMMA dissolution from the top of the CVD GR. The underside of the graphene layer is already clear and conducting, while the top side is initially coated with *ca.* 100 nm of insulating polymer (Fig. S3C†). In the case of a prior removal of PMMA before the metal nucleation, the full graphene surface is covered by the smaller NPs, as the whole upper area of the graphene is activated, so the NPs can nucleate everywhere (Fig. 4). By contrast, when the PMMA dissolution is carried out at the same time as the metal deposition, the localised nature of the deposition process allows larger structures to form at the grain boundaries (Fig. 3B–C). In both cases deposition was initiated at the topographically higher grain boundaries rather than the lower located planes.

2 AuCl_4_^–^ (aq) + 3DecMFc (o) → Au (s) + 4Cl^–^ (aq) + 3DecMFc^+^ (o)

**Fig. 5:**
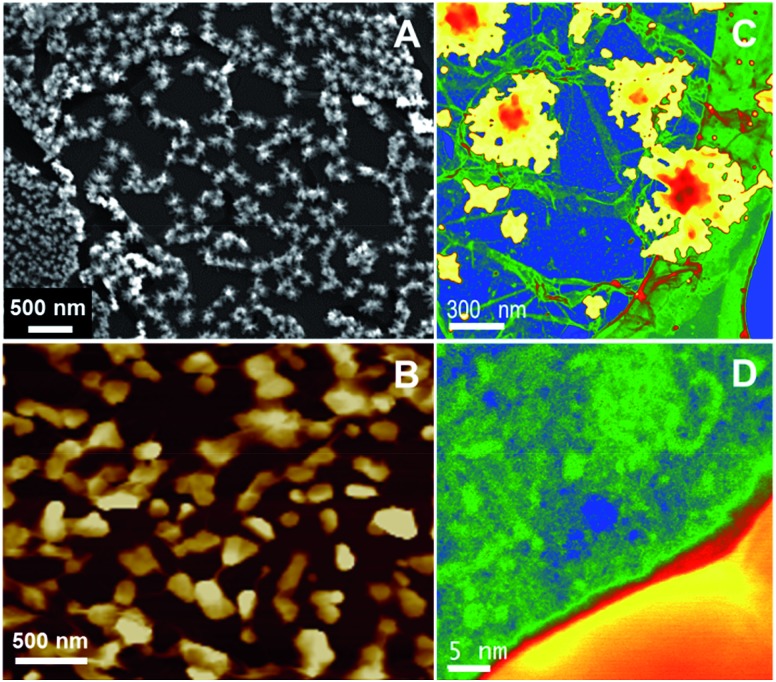
SEM (A), AFM (B) and STEM (C and D) images of the graphene-based gold nanoclusters shown in false colour and with the gamma value adjusted to allow the simultaneous visualisation of both Au and C (the relative intensities are therefore not representative of the usual *Z*-contrast of HAADF images). The interfacial contact time for deposition was 1 min. The DCE/5-nonanone ratio was 1 : 4 in the organic phase.

An analogous redox process can be applied to reduce tetrachloroaurate, AuCl_4_^–^, from the aqueous phase at the interface-assembled CVD GR (reaction 2). The resultant micrographs (SEM and AFM) are given in Fig. 5A–B. The smaller Au NPs seem to deposit uniformly on the graphene surface on the scale of the SEM (Fig. 5A), while AFM reveals (Fig. 5B) that particles in the range of 100–200 nm are deposited on the grain boundaries, the lines can be seen between the NPs. Aberration-corrected scanning transmission electron microscope (STEM) was employed to study the atomic composition with high-angle annular dark field (HAADF) imaging. The microscope was operated in “Gentle STEM” conditions at 60 kV as described elsewhere, in order to prevent any beam-induced damage to the graphene layer.^53,54^ Gold “nano-islands” on the graphene layer can be seen in Fig. 5C–D, which is shown in false colour and with its gamma adjusted to allow the simultaneous visualisation of the heavy Au islands (yellowish) and very light graphene support (blue), the original coloured STEM-HAADF images can be found in the ESI-5 (Fig. S6†). Fig. 5D shows a higher magnification HAADF image of the side of a bright Au “nano-island” (50–100 nm size scale), with Au lattice fringes just visible on the right-hand side. The darker blue and light green parts on the HAADF image (Fig. 5D) correspond to the monolayer graphene (see more in ESI, Fig. S2D†). The slightly brighter green features seen on the graphene surface are likely to be hydrocarbon based contamination along with heavier impurity atoms *i.e.* Si contaminants from the CVD process, invariably seen at the surface of freestanding graphene samples.^54,55^ Further elemental analysis was carried out using energy loss spectroscopy (EELS), revealing spectra characteristic of highly-graphitic carbon (graphene) and gold, are shown in the ESI-5.†

Given that the same electron donor is used in both cases, the thermodynamics of metal deposition are controlled solely by the reduction potentials of the metal species, +0.591 V and +1.002 V *vs.* standard hydrogen electrode, for the PdCl_4_^2–^/Pd and AuCl_4_^–^/Au couple, respectively.^56,57^

**Fig. 6:**
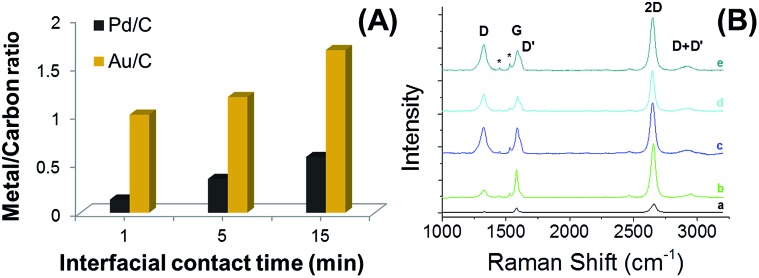
The relative metal amount on the decorated graphene as a function of contact time of deposition (A). Raman spectra (633 nm) of the pristine CVD GR (B-a), the Pd-CVD GR (B-b) and Au-CVD GR (B-c, d and e) nanoclusters, after 1 min (c), 5 min (d), 15 min (b and e) interfacial contact time. The DCE/5-nonanone ratio was 1 : 4 in the organic phase.

Increasing the deposition time in the case of Pd and Au (1 min, 5 min, 15 min) increased the number of the NPs and nucleation began on the graphene basal plane. After 15 min larger nanostructures were formed mainly on the grain boundaries, where the nucleation started (see ESI-6†). The metal content was characterized by energy dispersive X-ray analysis (EDAX), *via* the metal/carbon ratio as a function of the contact time, which is presented in Fig. 6A. The time dependence of metal content with contact time is shown in the ESI (Fig. S7†). The Raman spectra (633 nm) of the pristine (a), Pd-CVD GR (b) and Au-CVD GR (c–e) deposited monolayer graphene are given in Fig. 6B. The Pd-CVD GR nanocluster (b, after 15 min) was measured using 1 mW power while the Au NPs coated layers (c–e) were recorded with only 0.1 mW power (c, after 1 min; d, after 5 min; e, after 15 min), but the measured intensity values are comparable, because of the surface-enhanced Raman scattering (SERS) effect from the Au-CVD GR.^51^ The D′ (1613.5 cm^–1^) and D + D′ (2924.1 cm^–1^) peaks appeared on the spectra of graphene-metal nanoclusters (b–e), where D′ denotes a weak disorder-induced feature and D + D′ requires another defect for activation of the combination of phonons with different momenta.^17,18^ In the case of the gold nucleation, the 2D band upshifts from 2645.9 cm^–1^ (pristine CVD GR) to 2647.1 cm^–1^ (c, after 1 min), 2650.8 cm^–1^ (d, after 5 min), 2657.1 cm^–1^ (e, after 15 min) and the width is increased from 30.4 cm^–1^ (pristine CVD GR) to an average value of 43.3 cm^–1^ ± 0.93 cm^–1^ and the defect density (as measured from *I*_D_/*I*_G_) also increases with the deposition time, indicating more charging interaction between the Au NPs and graphene lattice.

**Fig. 7:**
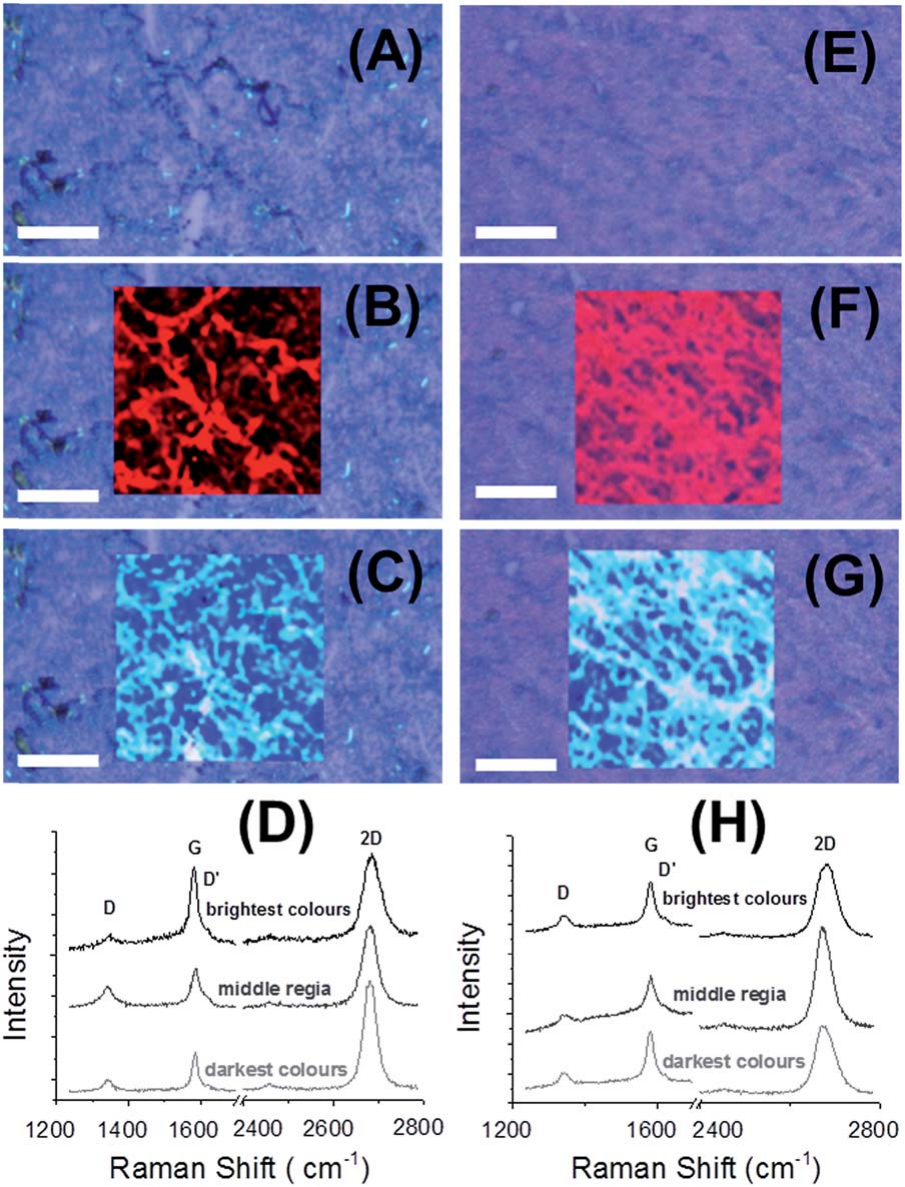
Optical micrographs of Pd (A–C) and Au (E–G) deposited CVD GR layer on Si/SiO_2_ substrate after 1 min deposition contact time (scale bar: 25 μm). The red highlighted squares (B and F) show the change of 2D width and the blue ones (C and G) display the change in the 2D position. Raman spectra of the Pd (D) and Au (H) NPs decorated graphene layers (532 nm), each an example from the different bright areas: brightest, middle, darkest colours (from the top). The interfacial contact time for deposition was 1 min in both cases. The DCE/5-nonanone ratio was 1 : 4 in the organic phase.

The spontaneous redox reaction, mediated by the GR, from the lower (aqueous) phase and the AFM images of the resultant CVD GR-metal nanoclusters suggested that the metal NPs nucleation has to take place on the underside of the graphene monolayer adsorbed at the ITIES (*i.e.* on the side in contact with the water) and, following transfer to the Si/SiO_2_ wafer, this side would lie between the graphene and solid substrate. The applied microscopic techniques, such as STEM, cannot give unambiguous direct evidence for the deposited metal NP position/location relative to the graphene single-layer, so to determine the metal NP location Raman spectroscopy, using 532 nm excitation light in mapping mode, was performed (Fig. 7A–H). In the case of Au^14^ or Ag^13^ NPs deposited on the upper side of the graphene, only the intensity ratio of the D and G bands changes, indicating an increase in defects from the interaction between the NPs and the graphene lattice.

Moreover, an intensity increase, by SERS, of a 4–5 nm thick thermally evaporated Au layer on mechanically exfoliated graphene has been reported.^51^ The positional shift of the Raman bands could come either from strain or doping of functionalized graphene, but a simultaneous increase (doping) or decrease (strain) of the FWHM of the 2D band provides a disambiguation between strain and doping.^8,9,17,51^ Fig. 7 shows the optical micrographic images of the Pd (Fig. 7A–C) and Au (Fig. 7E–G) NP-decorated CVD GR layers (after 1 min deposition), three Raman spectra (Fig. 7D and H) from each bright colour of the highlighted area (brightest, middle, darkest). The red and blue highlighted squares depict the width and the position of the 2D peak, respectively. The brighter parts are the maximum values, so the brightest red parts show the widest 2D peak and the lightest/brightest blue parts represent the most upshifted 2D peak positions. The dark/black structures (Fig. 7A), the Pd NPs are the brightest area (Fig. 7B and C), so the 2D band is upshifted from 2675.2 cm^–1^ (pristine CVD GR) to 2683.9 cm^–1^ and its width is increased from 35.3 cm^–1^ (pristine CVD GR) to 46.4 cm^–1^. The same trend can be observed in the case of Au NPs decorated single-layer graphene, the most upshifted 2D peak is 2679.7 cm^–1^ and the width of this peak is 50.9 cm^–1^, while from the bright parts a more uniform pattern and *ca.* 10-fold intensity increase due to the SERS effect were observed, as the Au NPs were deposited over the graphene surface (see ESI-6†). The defect density (measured from *I*_D_/*I*_G_) varies with location: the average value is 0.48 ± 0.12 for Pd and 0.68 ± 0.06 for Au NPs decorated CVD GR, which is almost twice the mean ratio of the pristine graphene. From the Raman band upshifts, the *I*_D_/*I*_G_ increase and the increase of the 2D width, it is deduced that the Pd and the Au NPs/nanostructures were deposited on the underside of the CVD GR layer so, after the transfer to Si/SiO_2_ wafer, the NPs are located between the single-layer graphene and the substrate.

### Electrochemically deposition of Pd NPs on adsorbed CVD GR

**Fig. 8:**
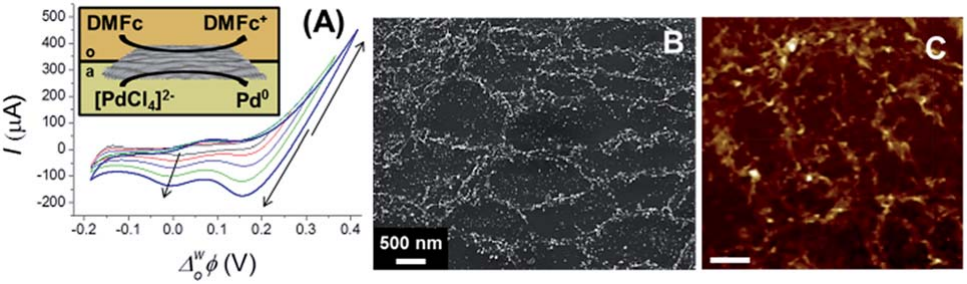
Cyclic voltammograms and schematic (A) of the electrochemical deposition of Pd NPs at interfacial assembled CVD GR, the SEM (B) and AFM (C, scale bar: 1 μm) images of the palladium decorated monolayer graphene. The DCE/5-nonanone ratio was 1 : 1.

The driving force of the above deposition reactions is a spontaneous redox process between the metal precursor and the organic phase electron donor. Alternatively an applied potential can be used to prepare CVD GR-metal nanoclusters *via* the ITIES approach. A complicating factor with Au is that the precursor AuCl_4_^–^ transfer from aqueous solution to the organic phase can be controlled through the applied potential,^36^ so in this case cyclic voltammetry was used only for the deposition of Pd NPs on the interfacially-assembled graphene single-layer (Fig. 8A). The supporting electrolytes were 0.1 M LiCl and 10 mM BTPPATPBCl in the aqueous and organic phases, respectively (see ESI-1†). In order to calibrate the whole potential window and the half-wave potential (*Δ*wo*Φ*_1/2_) scale, the tetraphenylarsonium tetraphenylborate (TATB) extra-thermodynamic assumption was used.^58^ The palladium deposition, from aqueous solutions of PdCl_4_^2–^, was induced by electron transfer from a less reactive organic phase electron donor, 1,1′-dimethylferrocene, DMFc (see schematic in Fig. 8A). The PMMA was dissolved before the experiments (following the procedure described above, DCE/5-nonanone ratio of 1 : 1), the voltammograms for Pd nucleation on CVD GR were carried out over different potential ranges, the increase of the current shows the nucleation (highlighted by arrows on Fig. 8A). After the electrochemical preparation, the samples were again transferred to the Si/SiO_2_ wafer. The SEM and AFM images of Pd decorated CVD GR are shown in Fig. 8B–C, the bright structures show the deposited metal particles, mainly on the grain boundaries (the topologically higher structures). The size of the Pd NPs underneath the CVD GR monolayer is in the 50–100 nm range, with an atomic (Pd/C) ratio of 0.19. In comparison to previously published papers on the noble metal thin film deposition at the oil/water interface-assembled reduced graphene-oxide flakes,^15,39–41^ the advantages of the present graphene modification are the ease with which mechanical assembly of the CVD GR single-layer at the ITIES is achieved and the grade of the high purity CVD graphene^5^ instead of the high defect density of reduced graphene-oxide flakes.^15^

## Conclusions

In summary, a very simple process to assemble low defect density CVD GR monolayers at a liquid/liquid interface, by direct transfer using PMMA on top of the monolayer, is demonstrated. The single-layer graphene sheet induces changes in the roughness of the interface, as detected through the halving of the interfacial capacitance detected using impedance spectroscopy, showing that the interfacial structure was stabilized by the monolayer graphene.

A simple spontaneous reduction process was applied to prepare graphene-based noble metal nanoclusters at the ITIES. In the case of the PMMA dissolution from the top of the CVD GR, two different kinds of deposited metal nanostructures were found: in the case of prior removal of PMMA before the metal nucleation, the full graphene surface was covered by smaller NPs (20–40 nm size range), while when PMMA dissolution proceeded at the same time as the metal deposition, larger nanostructures (40–300 nm range) were formed, and in both cases deposition was initiated at the grain boundaries rather than the planes.

The increase of the FWHM of the 2D Raman band from 30.4 cm^–1^ to 42.1–50.9 cm^–1^ reflects the doping effect of the metal NPs on the sp^2^ carbon lattice and the shift of the D, G and 2D peak positions is indicative of strain on the graphene. The combination of these changes by mapping mode Raman spectroscopy reveal the actual location of the metal NPs/nanostructures, underneath the CVD GR single-layer, providing direct proof of the deposition process: the NPs were grown from the lower (aqueous) phase under the graphene monolayer, which is adsorbed at the organic/water interface.

Furthermore, along with the spontaneous deposition on the ITIES assembled CVD GR, we have presented evidence of an electrochemically-controlled process, where the metal NPs were primarily deposited at the grain boundaries and thereafter on the planes.

The work reported opens up future, new vistas for graphene modification including the use of *in situ* Raman spectroscopy to study deposition on graphene at the liquid/liquid interface, use of the locus between two distinct solution phases to achieve the asymmetric functionalization of the graphene layer, and the exploration of the mechanism of stabilization of the monolayer graphene at the liquid/liquid interface.
