# Supplementary material for: Functionalization of graphene at the organic/water interface
Source: Chem Sci. 2014 Nov 25;6(2):1316–23. doi: 10.1039/c4sc03504f (PMC5811094; doi:10.1039/c4sc03504f)
Supplement: Supplementary file 1 [file SC-006-C4SC03504F-s001.pdf]

Electronic Supplementary Material (ESI) for Chemical Science.
This journal is © The Royal Society of Chemistry 2014

Supporting Information:

Functionalization of Graphene at the Organic/Water Interface

Peter S. Toth,[a] Quentin M. Ramasse,[b] Matěj Velický[a] and Robert A. W. Dryfe*[a]

[a]School of Chemistry, University of Manchester, Oxford Road, Manchester M13 9PL, UK

[b]SuperSTEM Laboratory, STFC Daresbury Campus, Daresbury WA4 4AD, UK

*E-mail: robert.dryfe@manchester.ac.uk, Tel: +44(0)161 306 4522

Supporting Information Abstract: These additional results and discussion presented in the Supporting Information include the four-electrode "inverse" liquid|liquid cell configuration and the cell compositions of voltammetry and impedance spectroscopy (S1), the microscopy characterization (optical, SEM, AFM, STEM) of the CVD GR transferred on Si/SiO_2 wafer or TEM grid (S2), AFM of the pristine and the PMMA spin-coated graphene monolayer on the solid substrate (S3), the photographs of the Pd deposition at the interface without and with the assembled CVD GR as a function of the interfacial contact time of the deposition (S4), the STEM images and EELS mapping of the Au metal NPs decorated CVD GR monolayer (S5), the SEM images and EDAX spectra of the deposited Pd or Au metal NPs at the graphene monolayer after different deposition times at the interface (S6).

S1 – The four-electrode inverse liquid│liquid cell configuration and the cell compositions of the voltammetry deposition and impedance spectroscopy

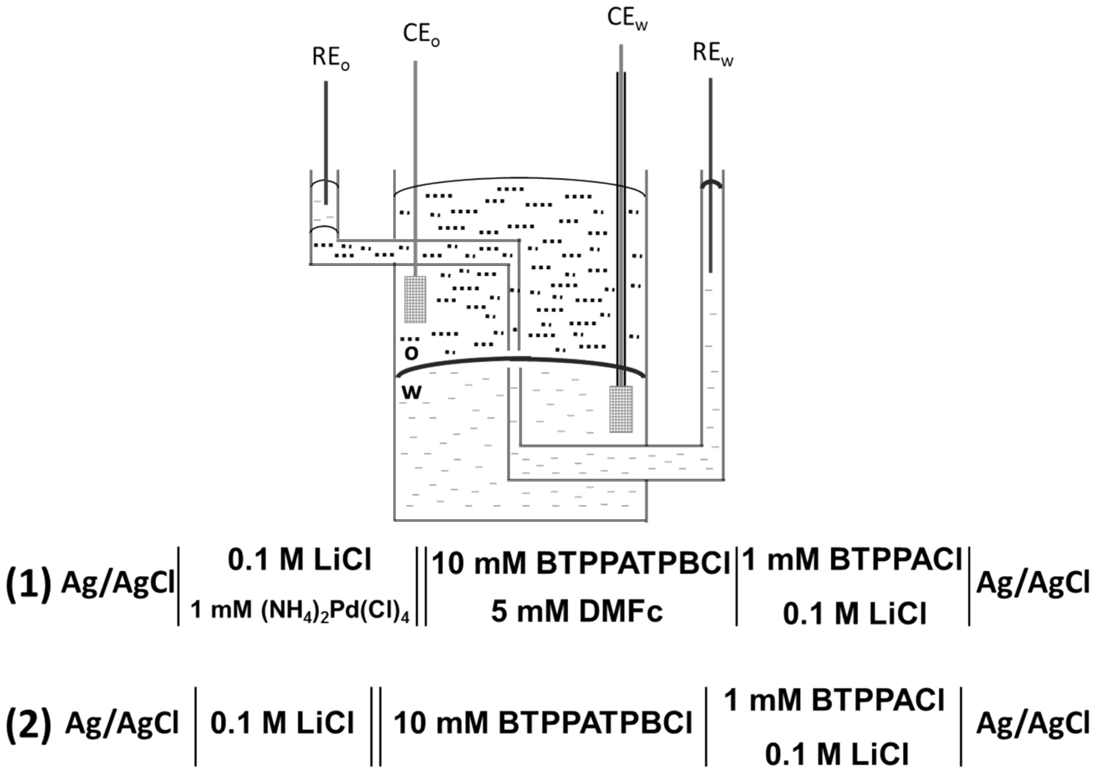

Figure S1 Schematic of the cell with the aqueous (w) and the organic (o) phases and the cell compositions of the voltammetry deposition (1) and impedance spectroscopy (2).

The cyclic voltammetry and electrochemical impedance spectroscopy (EIS) experiments were performed using a four-electrode configuration (Fig S1). Homemade Ag/AgCl reference electrodes (RE) were directly immersed in the chloride containing aqueous phase, an aqueous solution of 0.1 mM LiCl and 1 mM BTPPACl was brought in contact with the organic solution and formed a liquid junction for the organic reference electrode (RE_o). The aqueous counter electrode (CE_w) was glass coated to avoid contact of the Pt with the organic (upper) phase. The cell compositions (Fig S1) of the Pd NPs deposition using cyclic voltammetry (1) and for the EIS measurements (2) are shown.

S2 – Microscopic characterisation of the CVD GR on the Si/SiO_2 and lacey carbon coated copper grid substrates

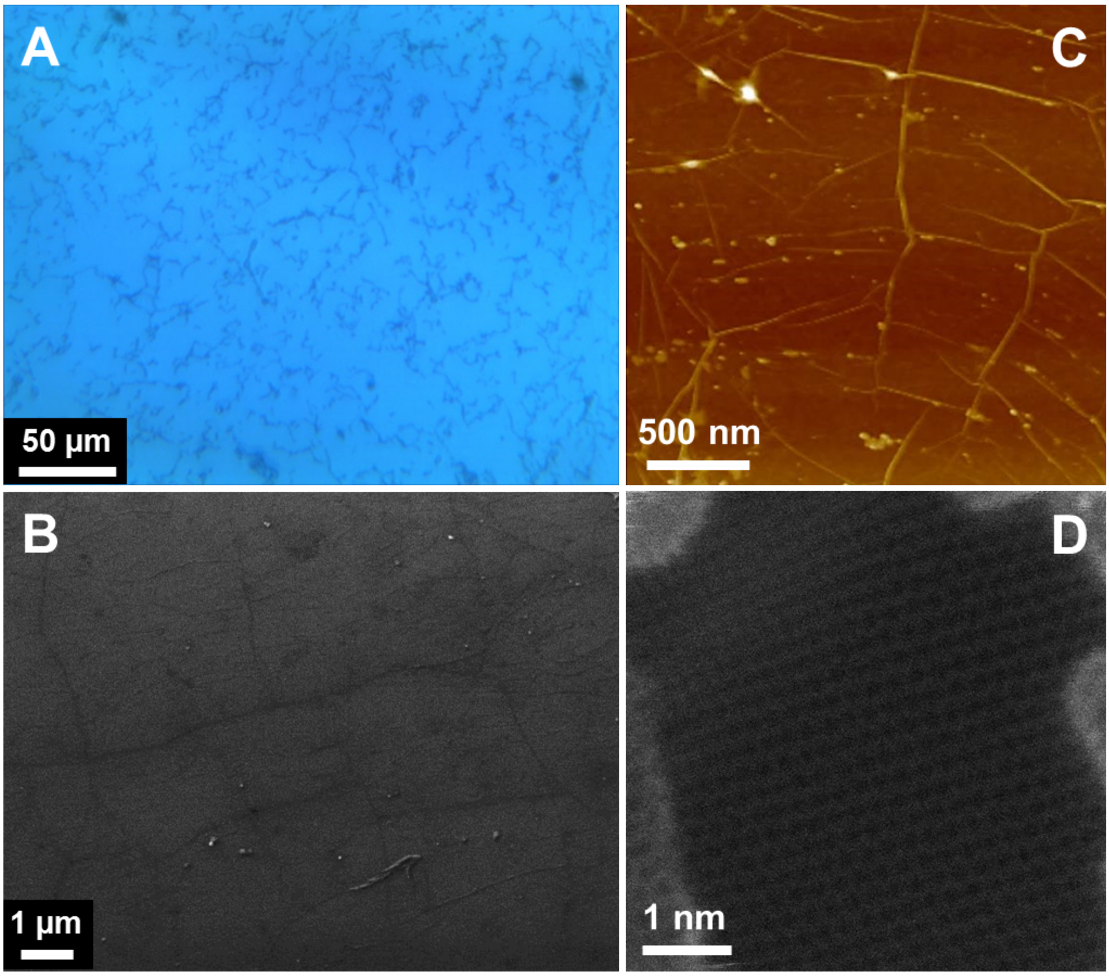

Figure S2 Optical micrograph (A), SEM (B), AFM (C) and atomic resolution HAADF (from consecutive scans) images from the pristine graphene monolayer on Si/SiO_2 or lacey carbon coated copper grid substrate.

The optical microscopic image (Fig S2A) shows the monolayer with the ridges/wrinkles/grain boundaries, which can be seen on the scanning electron microscopic (SEM) image (Fig S2B) and on atomic force microscopic (AFM) image (Fig S2C) as well. The ridges and wrinkles are underneath a continuous graphene monolayer, the CVD GR is visible only *via* these wrinkles and the graphene surface is dominated by these nano-sized ridges, which are 2–10 nm in height.[1-3] High-angle annular dark field (HAADF) imaging (Fig S2D) in the scanning transmission electron microscope (STEM) shows at atomic resolution the 2-dimensional single layer carbon atom honeycomb lattice, representing the *ca.* 1.42 Å length bonds between the carbon atoms.[4-6] The slightly brighter parts on the edge of the image are due to the presence of surface contamination on the graphene layer - likely hydrocarbon-based, which is ubiquitous in most free-standing graphene layers.[7]

S3 – AFM measurement of thickness of the graphene monolayer on the Si/SiO_2 substrate

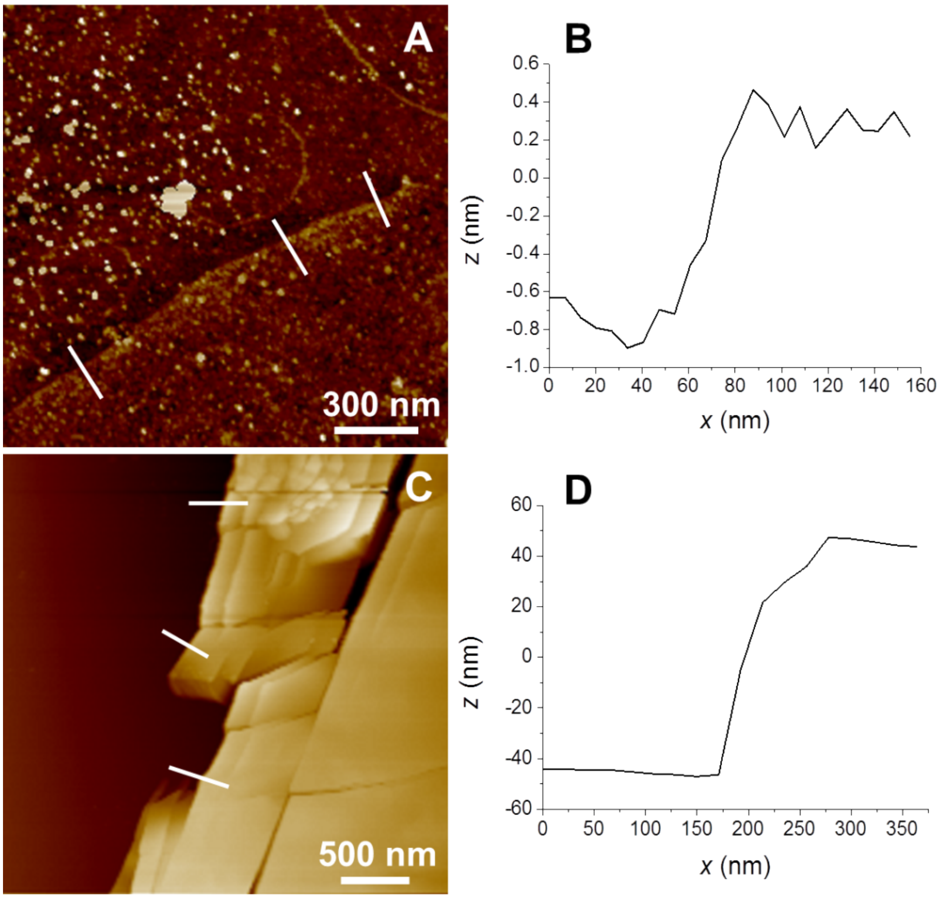

Figure S3 A: AFM image of the graphene layer on the Si/SiO_2, the bottom part is the graphene layer. B: The average height profile from cross sections (lines marked in A) between the layer and the substrate. C: AFM image of PMMA coated graphene layer on the Si/SiO_2. D: The average height profile from cross sections (lines marked in C) between the PMMA/CVD GR multilayers and the substrate.

Atomic force micrograph of the graphene layer is presented in Fig S3A, part of the monolayer is visible in lower part of image and Fig S3B shows the average height profile from cross sections along the edge (marked with lines in Fig S3A) between the GR layer and the substrate. The step height of the GR layer is *ca.* 1 nm, which corresponds to monolayer, in reasonable agreement with the known value for pristine monolayer graphene (0.35 to 1 nm).[3,8] The additional particles of several nanometers in height are residues of the poly (methyl methacrylate) (PMMA) polymer that was used to transfer the graphene to the Si/SiO_2 substrate.[3] More particles can be seen on the Si/SiO_2, as these are moved from the top of the graphene layer during the PMMA dissolution, moved and remained on the substrate. Figure S3C displays the PMMA spin-coated CVD graphene (PMMA/CVD GR multilayer), the PMMA is the yellowish right part and the average height profile from cross sections along the edge (marked with lines in Fig S3C) between the PMMA/CVD GR multilayer and the Si/SiO_2 is depicted by Figure S3D. The step height of the multilayer is *ca.* 100 nm.

S4 – Photographs of Pd NPs deposition at ITIES and the interface-assembled CVD GR as a function of contact time

Figure S4 shows the photographs of the spontaneous metal NPs deposition reaction as a function of the contact time (A: 1 min, B: 5 min, C: 15 min, D: 30 min) at the interface between two immiscible electrolyte solutions (ITIES). The first vial shows the PMMA/CVD GR multilayer at the interface, the graphene monolayer becomes invisible after a while due to the PMMA layer dissolution. The second vial shows the palladium salt ($(NH_4)_2PdCl_4$) in the aqueous phase (down) and the DecMFc reducing agent in the organic solution (upper). The third vial contains the same concentrations of the reactant species as the second one and the graphene layer is assembled at the interface. The darker Pd NPs decorated graphene single-layer can be observed after 1 min (A), while without the graphene, in the middle vial nothing can be seen. Only after 15 min (C) is a darker "layer" (Pd NPs) visible, which becomes thicker after 30 min (D), so with the CVD GR at the interface the spontaneous nanoparticle deposition is faster than without.

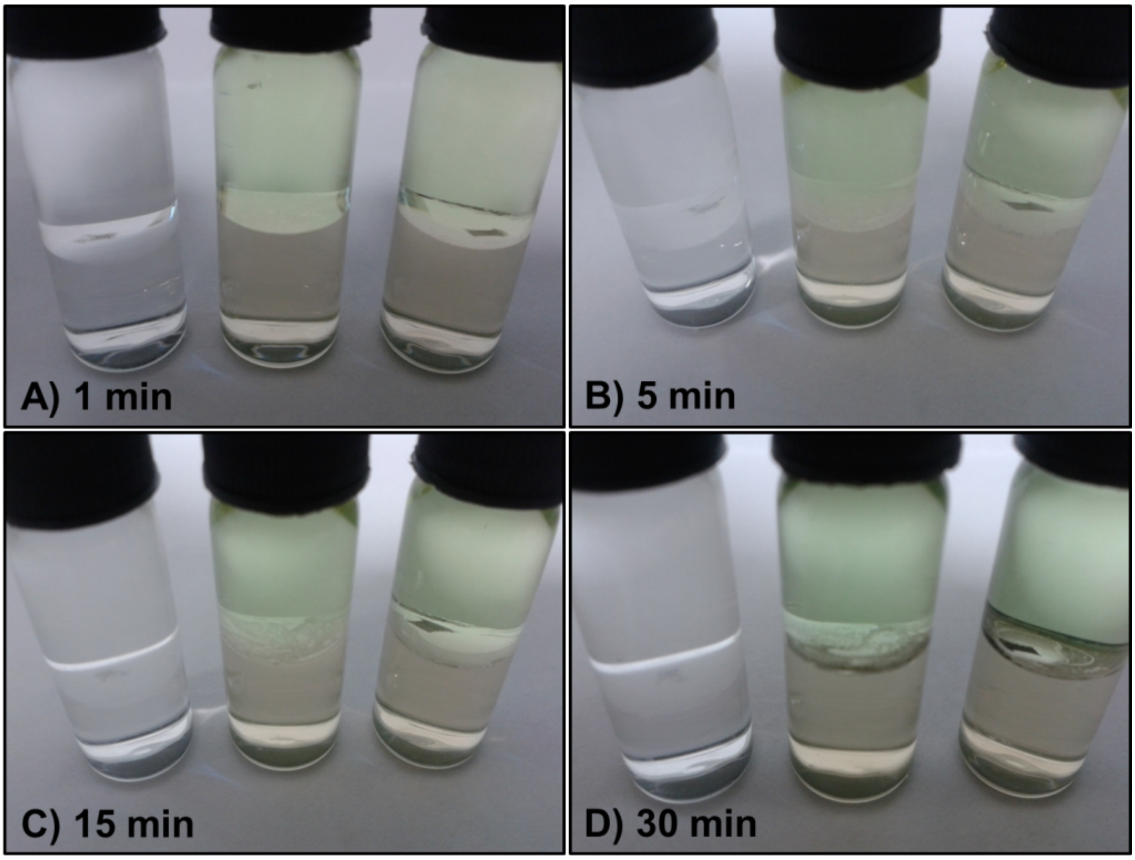

Figure S4 Photographic images of the Pd NPs deposition at the bare interface and at the interface assembled CVD GR (≈ 5 mm × 7 mm) after 1 min (A), 5min (B), 15 min (C) and 30 min (D) contact time. The first vial contains the supporting electrolyte in both phases and graphene monolayer at interface, in the middle one there are the supporting electrolytes and 1 mM $(NH_4)_2PdCl_4$ in the aqueous solution, 2 mM DecMFc in the organic phase, while in the third vial there is the same composition than the second one and the CVD GR is assembled at the interface. The DCE/5-nonanone ratio is 1:4.

S5 – STEM images and EELS mapping of the Au metal NPs decorated CVD GR monolayer

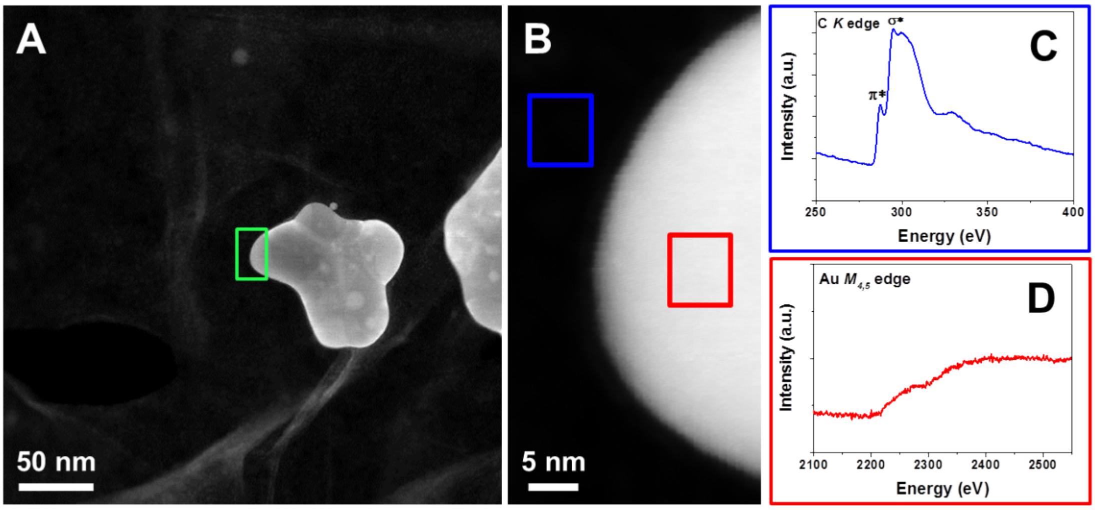

Figure S5 HAADF (from consecutive scans) images (A, B) from the deposited Au "nano-island" on graphene monolayer (the structure was then transferred to a lacey carbon coated copper grid for electron microscopy observation). B is a magnified view of the region highlighted by a green rectangle in A. C-D: EELS spectra acquired from the areas marked by the squares in B. The carbon *K* edge spectrum observed in C, and its near-edge fine structure - the marked σ* and π* peaks in particular, are characteristic of graphitic carbon, confirming the presence of graphene. Furthermore, the clear Au $M_{4,5}$ signal observed in D confirms the identification of the particle as a Au island. The interfacial contact time of deposition was 1 min. The DCE/5-nonanone ratio was 1:4 in organic phase.

Figure S5A shows a STEM-HAADF image of a Au metal "nano-island" on CVD GR. Electron energy loss spectroscopy (EELS) spectrum imaging, whereby EEL spectra are acquired serially at each pixel across the area defined by the green rectangle, was used for elemental analysis to unambiguously confirm the presence of graphene and gold particles.[9] Example spectra, extracted from the areas marked by red and blue squares on Fig S5B, which shows the HAADF intensity acquired simultaneously with the EELS spectrum image, are presented in Fig S5C-D. The spectra are extracted from two datasets acquired consecutively from the very same area, but with different energy ranges to capture both the C *K* edge and Au $M_{4,5}$ edges. The EELS data was denoised using principal component analysis. The observed C *K* edge does not correspond to a typical spectrum from pure single layer graphene due to the presence of a layer of carbon-based contamination on the surface, but it retains very clear graphitic characteristics confirming the presence of an underlying graphene layer (as clearly seen on Fig S2D). Figure S6A-B depict the original STEM-HAADF images of graphene-based gold nanoclusters which are shown in Figure 5C-D (in the main paper). (By "the original" we mean the original colour without the gamma adjustment.)

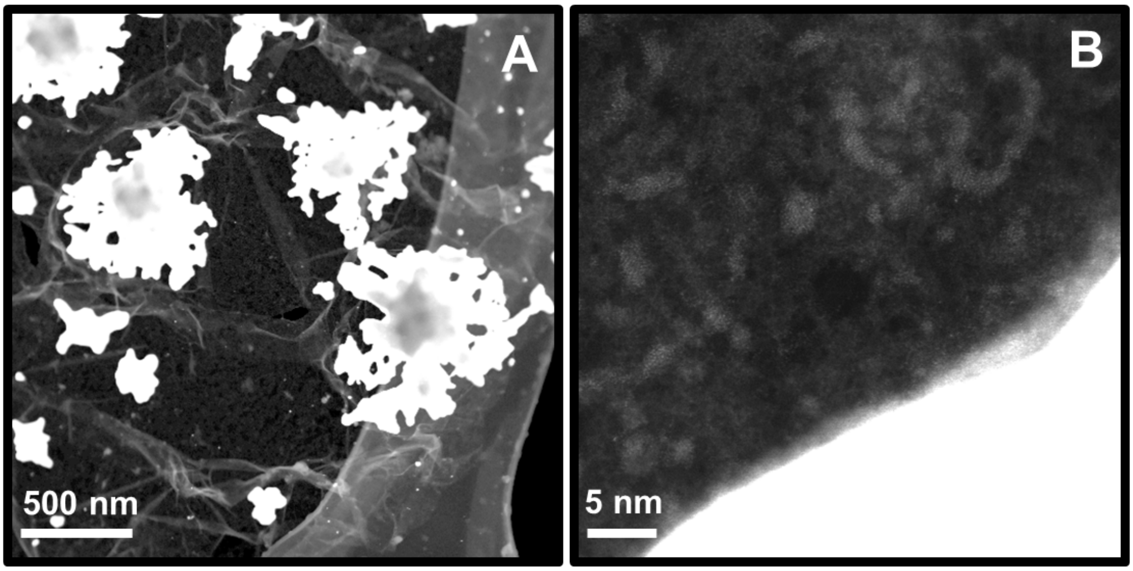

Figure S6 The original STEM-HAADF images of the graphene-based gold nanostructures from Figure 5. Figure S6A is the original of Fig 5C and Figure S6B is the original of Fig5D.

S6 – SEM images of the deposited Pd and Au metal NPs on the graphene monolayer after different contact times at the interface

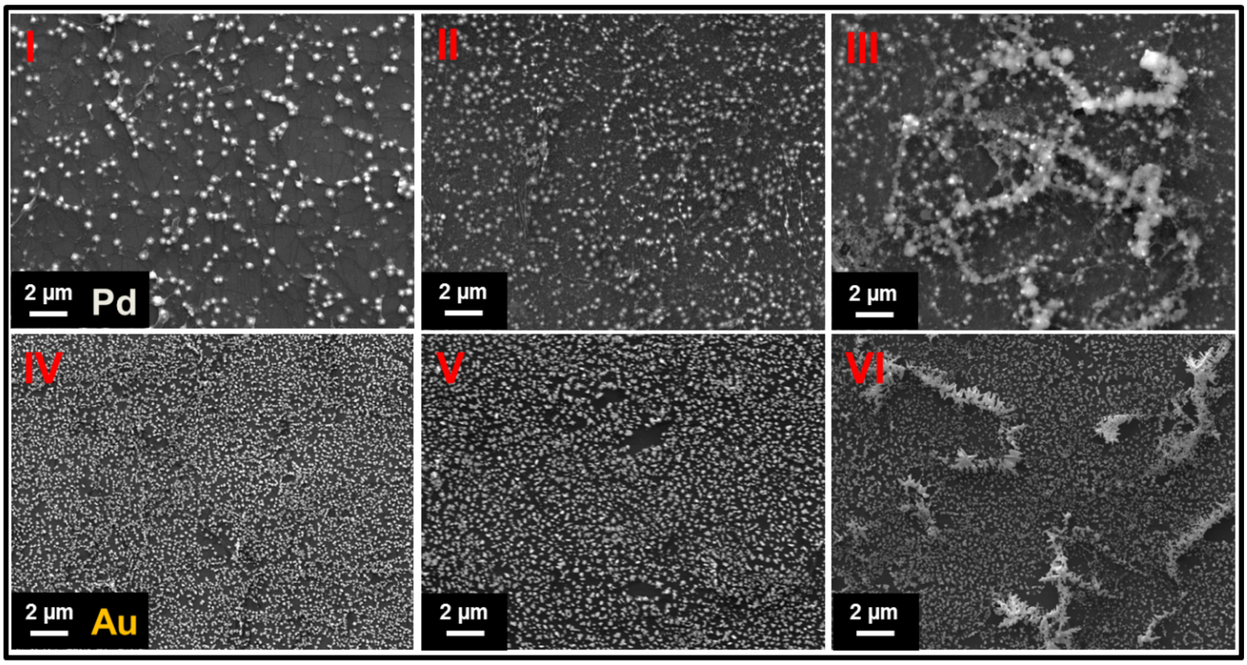

Figure S7 SEM images of Pd (I, II, III) and Au (IV, V, VI) NPs and nanostructures at graphene monolayer after 1 min (I, IV), 5 min (II, V) and 15 min (III, VI) interfacial contact time of deposition. The DCE/5-nonanone ratio was 1:4 in the organic phase.

Figure S7 shows the SEM images of the globular shaped metal NPs at CVD GR in the case of larger palladium (Fig S7 I, II, III) and in the case of smaller gold NPs (Fig S7 IV, V, VI), samples were transferred on Si/SiO_2 wafer. The amount of the metal particles, the number of the NPs and the formed larger nanostructures increases as a function of the interfacial contact time of deposition, after 1 min (I, IV), 5 min (II, V), 15 min (III, VI). Figure S8 presents the energy dispersive X-ray (EDAX) spectra of the deposited metal NPs at CVD GR in the case of palladium (Fig S8A) and in the case of gold (Fig S8B) on Si/SiO_2 wafer.

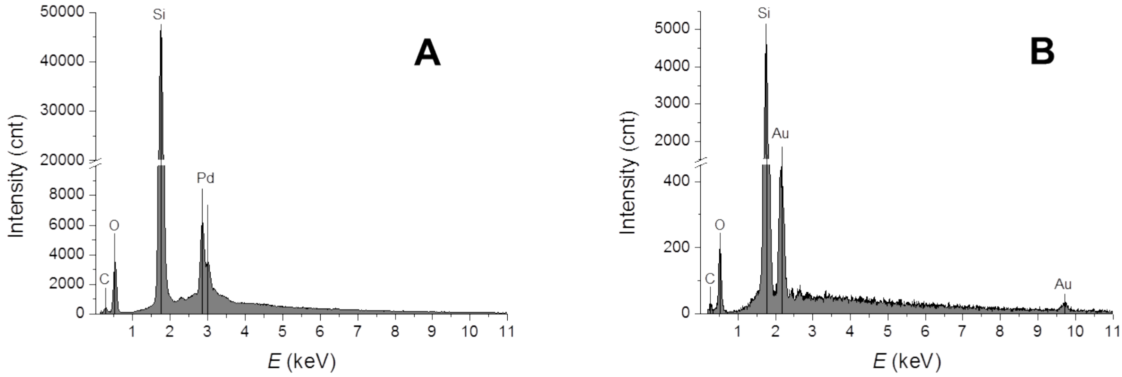

Figure S8 The EDAX spectra of Pd (A) and Au (B) NPs coated graphene monolayer on the Si/SiO_2 wafer.

REFERENCES

1. X. Li, W. Cai, J. An, S. Kim, J. Nah, D. Yang, R. Piner, J. Velamakanni, Jung, E. Tutuc, S. K. Banerjee, L. Colombo and R. S. Ruoff, *Science*, 2009, **324**, 1312-1314.
2. X. An, F. Liu, Y. F. Jung and S. Kar, *J. Phys. Chem. C*, 2012, **116**, 16412-16420.
3. S. Zhao, S. P. Surwade, Z. Li and H. Liu, *Nanotechnology*, 2012, **23**, 355703 (6pp).
4. J. H. Warner, G. D. Lee, K. He, A. W. Robertson, E. Yoon, and A. I. Kirkland, *ACS Nano*, 2013, **7**, 9860–9866.
5. G. Casillas, A. Mayoral, M. Liu, A. Ponce, V. I. Artyukhov, B. I. Yakobson and M. J. Yacaman, *Carbon*, 2014, **66**, 436-441.
6. R. Zan, Q. M. Ramasse, U. Bangert and K. S. Novoselov, *Nano Lett.*, 2012, **12**, 3936-3940.
7. W. Zhou, M. P. Prange, M. P. Oxley, S. T. Pantelides, S. J. Pennycook, J. Nanda, C. K. Narula and J. C. Idrobo, *Microsc. Microanal.,* 2011, **17**, 1498-1499.
8. K. S. Novoselov, A. K. Geim, S. V. Morozov, D. Jiang, Y. Zhang, S. V. Dubonos, I. V. Grigorieva and A. A. Firsov, *Science*, 2004, **306**, 666-669.
9. Q. M. Ramasse, R. Zan, U. Bangert, D. W. Boukhvalov, Y. W. Son and K. S. Novoselov, *ACSNano*, 2012, **6**, 4063-4071.
